# Clinical application of myofascial therapy in the treatment of postpartum pain-related functional disorders: A review

**DOI:** 10.1097/MD.0000000000039869

**Published:** 2024-10-04

**Authors:** Jiangchun Zhang, Tingting Pang, Junjie Yao, Ailin Li, Li Dong, Yueting Wang, Yufeng Wang

**Affiliations:** a College of Acupuncture and Tuina, Changchun University of Chinese Medicine, Changchun, China; b The Rehabilitation Medicine Department of Zhejiang Provincial People’s Hospital, China.

**Keywords:** biomechanics, myofascial therapy, postnatal recovery, postpartum dysfunction, postpartum pain

## Abstract

During pregnancy, fetal growth could lead to changes in human biomechanics. If postpartum recovery was not properly managed, it could be exacerbated, resulting in myofascial system disorders and various functional impairments. Among them, pain-related functional disorders were an important issue affecting quality of life in postpartum women. The pathogenesis of these disorders remained unclear but it was primarily associated with changes in biomechanics, the endocrine system, and nervous function. However, postpartum pain-related dysfunction had been considered a normal physiological response to childbirth, leading to a lack of attention. Therefore, many postpartum women failed to receive timely, effective, and standardized treatment, hindering their ability to reintegrate into family and society, and causing severe damage to their physical and mental health. In clinical practice, myofascial therapy could effectively alleviate postpartum pain and muscle spasms, improve excessive tension injuries in myofascial, and had a good therapeutic effect on postpartum pain-related functional disorders. The mechanism of myofascial therapy involved improving core muscle strength, restoring normal body alignment, and promoting the remodeling of myofascial mechanical structures. This article explored the positive effects of myofascial therapy on postpartum pain-related functional disorders from a biomechanical perspective, aiming to provide diverse treatment approaches for clinical practitioners.

## 1. Introduction

During pregnancy, women experienced various physiological and anatomical changes, with biomechanical changes being the most pronounced and becoming more significant as the fetus grows. Usually, these changes returned to normal levels after delivery. However, inadequate postpartum care could lead to persistent mechanical alterations, resulting in musculoskeletal pain, pelvic misalignment, Pelvic floor dysfunction (PFD), diastasis recti, and sacroiliac joint disorders. Multiple studies had shown that more than 50% of mothers would have varying degrees of dysfunction,^[1,2]^ with this proportion potentially being higher among first-time mothers.^[3]^ These dysfunctions could significantly impact the quality of life and might even lead to postpartum depression.

During breastfeeding, the mother should consider complementary and alternative therapies to avoid the indirect effects of chemical drugs on the baby. Myofascial therapy was a targeted treatment based on the myofascial chain theory, focusing on biomechanical changes. This therapy involved external mechanical stimulation by the therapist to adjust the disordered myofascial system and restore it to the correct biomechanical pattern.^[4]^ In the clinic, the therapy had demonstrated efficacy in alleviating postpartum pain-related dysfunction in women. It was widely accepted due to its safety and lack of side effects.^[5]^ This article reviewed the application of myofascial techniques in managing postpartum pain and explored potential future developments. It aimed to offer a comprehensive diagnostic and therapeutic framework for the clinical treatment of postpartum pain, thereby contributing to the improvement of the quality of life for postpartum women.

## 2. Biomechanical changes during pregnancy and postpartum

A growing fetus induced a range of biomechanical changes in the mother, including postural adjustments influenced by lumbar and pelvic structures, altered gait patterns due to load changes and mechanical stress, and abnormal muscle activation patterns resulting from neuromuscular adaptive mechanisms.

### 2.1. Postural change

Postural changes during pregnancy and the postpartum period were closely related to hormone secretion and spinal curvature. Studies had found that relaxin, estrogen, and progesterone played an important role in the occurrence and intensity of pregnancy-related pain.^[6,7]^ These hormones could cause spinal and pelvic instability through the influence of relaxation of the peripheral ligaments, thereby inducing nonjoint damage and subsequent pain.^[8]^ A long-term follow-up study involving 29 healthy pregnant women found that pregnancy-induced multiplanar ligament relaxation in the knee joint, along with decreased backward compliance and increased forward compliance, persisting up to 5 months postpartum.^[9]^ In addition, hormone levels were associated with the production and remodeling of collagen in the joints. Studies had confirmed that estrogen and relaxin caused substantial loss of glycosaminoglycans and collagen in the symphysis pubis and temporomandibular joint discs, as well as collagen in articular cartilage.^[10]^ Collagen was closely related to the biomechanical properties of muscles, fascia, and ligaments. Reduced collagen content leaded to ligament damage and muscle strength imbalance, contributing significantly to postural changes. Additionally, changes in spinal curvature also played a role in altering posture. Observational studies had shown a tendency for decreased thoracic kyphosis, lumbar lordosis, and lateral deviation during pregnancy.^[11,12]^ These changes might lead to postpartum issues such as the Trendelenburg sign, knee valgus, lumbar lordosis, and other alignment disorders, as well as abdominal wall relaxation and flexibility issues in muscles such as the hip flexors, lumbar extensors, iliotibial band, and hamstrings.^[13]^

### 2.2. The gait changes

During pregnancy, changes in physiological morphology could affect the characteristics of female gait, and biomechanical analysis showed that pregnancy had the most significant effect on the dynamic joint variables of the hip, knee, and ankle.^[14]^ LI X found that during pregnancy, women’s walking speed, stride length, stride frequency, thigh acceleration, and leg falling force all decreased to varying degrees. Additionally, joint movement of the lower limbs decreased in the sagittal plane, and joint torque decreased.^[15]^ In addition, the reduction in article-induced acceleration when walking in the third trimester began to rely more on the gluteus maximus rather than the quadriceps to extend the knee joint. This shift potentially aimed to mitigate strong interactions between the pregnant woman and the ground, consequently reducing mechanical load. In pregnant women exhibiting pain symptoms, researchers observed decreased hip utilization and increased ankle torque and muscle activation during walking.^[16]^ The asymmetry of rotational and translational motion increased significantly, contrasting with healthy pregnant women who exhibited reduced gluteus maximus activation.^[17,18]^ These factors destroyed the lumbar and pelvic force closure and dynamic stability and increased the joint load. Altered gait patterns resulting from these factors might have led to impaired stability during walking in pregnant women, contributing to musculoskeletal pain during pregnancy and postpartum. Nonetheless, pregnant women could adapt their gait to enhance balance by adjusting stride length or frequency and stabilizing their center of gravity.^[19]^

### 2.3. Musculoskeletal change

During pregnancy, neuromuscular adaptive protection caused alterations in muscle activation patterns. Studies demonstrated that pregnant women exhibited changes in lumbar movement patterns and high activation of the erector spinal muscles during trunk flexion and extension. These changes prevented increased abdominal swelling and ligament relaxation through these biomechanical changes.^[20]^ While standing, women in the third trimester did not change their lumbar pelvic position but showed increased activation of the lumbar and pelvic extensor muscles, indicating an adaptive response of the trunk extensor muscles to increased abdominal volume.^[21]^ After lumbar stability and flexibility training in pregnant women with low back pain (LBP), the degree of activation of trunk active muscles was significantly increased, and pain and balance were also improved.^[22]^ The alteration in muscle nerve activation is suggested to be an important factor influencing pain during pregnancy. In addition, the reversibility of neuromuscular adaptations that occurred during pregnancy was also demonstrated, with comparable changes in trunk and lower limb muscle activation during upright and trunk flexion and extension movements before and after pregnancy.^[21]^ This indicates that progressive short-term neuromuscular adaptations during pregnancy could rapidly subside after delivery. In terms of bone changes, the metabolism of calcium and other minerals in the body changed significantly during pregnancy and breastfeeding. Throughout pregnancy, the body’s calcium and mineral metabolism must be adapted to the needs of the fetus and placenta to promote bone mineralization in the developing fetus. Analysis of iliac bone biopsies at the beginning and end of pregnancy showed that pregnancy significantly altered the metabolic status of maternal bone.^[23]^ During breastfeeding, lactating women experienced increased calcium loss when producing milk. If the body failed to absorb sufficient calcium to meet this demand, it mobilized calcium from maternal bone by activating osteoclast activity or bone lysis mechanisms, leading to a decrease in bone mass. A study revealed that women who exclusively breastfed their babies exhibited heightened osteoclast activity, while osteoblast activity remained largely unchanged.^[24]^ This suggested that bones underwent a high turnover state during the postpartum period, and some pregnant women might have been susceptible to fragility fractures due to metabolic changes and mechanical stress induced by breastfeeding.

## 3. Clinical application of myofascial therapy

The myofascial membrane served as the conduction and support system of systemic tension, with local mechanical load distributed throughout the structure along the fascial tension lines. When the human body experienced local functional abnormalities, it would call upon other tissues to compensate for changes in tissue tension or stress, thus maintaining overall stability. While adjacent tissues possessed a certain compensatory capacity, they frequently generated various pathological reactions due to excessive compensation. Throughout pregnancy, women experienced significant mechanical changes that resulted in extensive transitional compensation. The survey revealed that nearly all pregnant women experienced pain and dysfunction resulting from mechanical changes, with approximately 25% experiencing temporary disabling symptoms.^[25,26]^ Moreover, inappropriate treatment could lead to postpartum fatigue, pain, muscle tension, and physical discomfort without organic damage. Therefore, mastering the clinical indications and treatment methods of myofascial therapy was crucial for enhancing the quality of life of women during pregnancy and postpartum.

### 3.1. Postpartum low back pain

More than 70% of pregnant women suffered from pregnancy-related LBP.^[26]^ Pregnancy-induced changes in the fascial system were characterized by contraction of the rectus femoris muscle, elongation of the rectus abdominis muscle in the anterior fascial line, contraction of the hamstring muscles in the posterior fascial line, and contraction of the erector spinae muscles. These changes resulted in an imbalance of myofascial tension and increased myofascial irritability. Any part of the muscles associated with the lower back and pelvis could activate the myofascial pain trigger, leading to LBP.

Ożóg et al found that a single myofascial release technique effectively and sustainably reduced the resting activity level of erector and multifidus muscles in LBP patients, decreased pain intensity, and improved functional performance. The therapeutic effect was attributed to the manipulation of tissue tension in the thoracolumbar fascia and the enhancement of flexibility and sliding between soft tissue layers.^[27]^ He et al used fascia therapy to treat postpartum LBP. They selected coordination centers on different fascia sequences based on various symptoms. By pressing and pushing the trigger points of lumbar and back myofascial pain, they found that fascia therapy not only enhanced the stability of core muscles but also significantly reduced myofascial tension and alleviated pain symptoms.^[28]^ Schwerla et al used osteopathic therapy incorporating myofascial technology to treat 40 postpartum women with LBP 4 times. Compared with conventional treatment controls, osteopathic therapy showed more significant improvements in pain intensity and dysfunction indices.^[29]^ Forty postpartum women with LBP were treated 4 times with osteopathic therapy incorporating myofascial technology, and improvements in pain intensity and dysfunction indices were more significant with osteopathic therapy than with conventional treatment. Therefore, the application of myofascial therapy helped relieve postpartum LBP in women.^[30]^

Therefore, myofascial techniques aim to alleviate it by correcting improper mechanical structures around the pelvis. Initially, these techniques focus on restoring proper alignment of the pelvis in 3 dimensions by improving the relationships between muscles and fascia. Once structural alignment is achieved, pain symptoms typically subside. Subsequently, comprehensive rehabilitation of the 4 myofascial chains surrounding the pelvis is necessary to reinforce pelvic stability. This approach addresses the root cause of LBP, providing a long-term solution. However, existing clinical trials did not provide sufficient evidence to support the clinical use of this therapy, and highlighting an urgent need to harmonize terminology and optimize treatment protocols.^[31]^

### 3.2. Pelvic floor dysfunction

Pelvic floor dysfunction (PFD) was a common functional disorder observed frequently during pregnancy and after delivery, characterized by clinical manifestations such as pelvic organ prolapse, sexual dysfunction, defecation dysfunction, and pelvic pain. Studies had indicated that 20% of women developed some type of PFD during pregnancy, with 21% still experiencing residual symptoms 2 years after delivery.^[32]^ In a study involving 200 postpartum women, Yang et al discovered that the incidence of PFD in parturients was significantly higher compared to women who gave birth vaginally.^[33]^ Chen reported a higher probability of PFD in women who delivered naturally through the vagina compared to those who delivered via cesarean section. Additionally, pelvic floor muscle strength was lower in women delivering with forceps or fetal head suction compared to those delivering via cesarean section.^[34]^

One systematic review observed that women who underwent pelvic floor rehabilitation were 5 to 8 times more likely to eliminate symptoms of urinary incontinence compared to women who received general education, lifestyle advice, or no intervention.^[35]^ Clinical studies have further validated that myofascial therapy can alleviate PFD by reducing tension and pain sensitivity, enhancing the function of the endogenous inhibitory system, and addressing psychological issues such as anxiety, depression, somatization symptoms, and pain catastrophization.^[36]^ Additionally, DIEB et al implemented prenatal intervention through vaginal digital massage on mothers, effectively reducing perineal tearing and postpain.^[37]^ An et al discovered that manipulation of the pelvic fascia to relax pelvic floor muscles, combined with electrical stimulation therapy, could increase the surface myoelectric value of the pelvic floor, ameliorate maternal stress urinary incontinence, decrease the severity of pelvic organ prolapse, enhance pelvic floor muscle strength, and alleviate pelvic myofascial pain.^[38]^

From the perspective of the fascial chain, pelvic floor muscles and fascia underwent excessive elongation during pregnancy. Although the relaxed muscles gradually recovered after delivery, the elasticity of fascia was weaker than that of muscles, making it easy for fascial folds to increase and overlap in the inner thigh region. This led to issues such as heightened tension and irritability of pelvic floor fascia, thereby predisposing individuals to PFD. Myofascial techniques targeted at the pelvic floor have been observed to release trigger points responsible for pain, alleviate fascial contractures, reduce pain sensitivity, and relieve pelvic floor muscle spasms. These benefits can improve pelvic floor muscle function, expedite the recovery of pelvic floor structures, and ultimately decrease the occurrence of chronic pelvic pain and other PFDs. In future research, it is recommended to conduct more high-quality, standardized, and long-term follow-up studies to comprehensively understand the treatment effectiveness of this therapy for PFD, thereby providing sufficient data support for its clinical application.

### 3.3. Diastasis recti abdominis

Diastasis recti abdominis (DRA) occurred when fetal growth caused gradual distention of the female abdomen. Mechanical compression resulted in stretching and thinning of the abdominal white line, and gradual separation of the recti abdominis muscles on both sides.^[39]^ According to statistics, the incidence of DRA at 6 weeks, 6 months, and 12 months postpartum was 60%, 45.4%, and 32.6%, respectively.^[40]^ Although some literature studies had shown that there was no significant correlation between DRA and postpartum problems such as LBP and PFD^[40]^ serious DRA was related to changes in the fascial chain of the body surface. The internal and external oblique muscles in the bilateral anterior oblique chain were elongated, thereby affecting abdominal wall function and trunk stability, resulting in issues such as weakness, LBP, and more serious complications such as pelvic organ displacement, prolapse, hernia, and significantly reducing women’s quality of life.^[41]^

Currently, there were no effective clinical measures to prevent DRA, and studies had indicated that neither abdominal core training nor pelvic floor muscle training could effectively prevent or reduce the incidence of prenatal and postnatal DRA.^[42,43]^

Lishuang Tan et al discovered that the application of “wandering pot” on the fascia of the abdomen, chest, and waist effectively enhanced fascial extension, alleviated muscle tension and spasms, improved DRA status, and reduced abdominal circumference.^[44]^ Zhu et al, in their research, demonstrated that traditional Chinese massage on the abdominal fascia enhanced local blood circulation, reduced muscle stiffness, and when combined with electrical stimulation, stimulated abdominal muscle contraction, effectively ameliorating DRA and enhancing women’s quality of life.^[45]^ Yu et al undertook a comprehensive treatment approach for postpartum DRA using traditional Chinese medicine, incorporating anatomical, pathological, and traditional Chinese meridians theories. Their findings suggested that physical therapy relieved spasms in the external oblique muscle, alleviated separation tension, activated muscle strength and fascia tension in the transverse muscle, created displacement space behind the rectus sheath, and notably shortened the repositioning time of DRA.^[46]^

Based on the etiology of DRA, myofascial therapy was able to address the underlying issues by effectively treating the prolonged muscles and fascial tissues in the lumbar abdomen. This therapy stimulated peripheral nerve motor neurons, reactivated proprioceptive receptors in damaged muscles, enhanced muscle fiber contraction, and facilitated the restoration of normal elasticity in the elongated rectus abdominis. Myofascial techniques contributed to the restoration of the original length of stretched muscles and fascia, aiding in DRA recovery. Moreover, they reinstated the tension structure of the rectus sheath and improved the superficial fascia’s structure within the subcutaneous fat layer, thereby enhancing abdominal sensation and metabolism and laying the groundwork for subsequent fat layer reduction. Further research is imperative to explore the long-term efficacy and refine clinical interventions for the prevention and management of DRA.

## 4. Summary and prospect

In summary, myofascial system disorder is 1 of the important causes of postpartum pain-related dysfunction. Myofascial therapy can relieve postpartum pain and dysfunction by releasing soft tissue, resetting the correct biological force line, adjusting the structural balance of the body, improving subcutaneous tissue microcirculation, and accelerating the metabolism of inflammatory factors and pain-causing substances. However, there are still problems with the treatment in clinical research and application. First, due to the lack of standardized treatment methods and uniform quantification criteria of efficacy, it is currently impossible to effectively conduct multi-center clinical studies and implement high-quality trial control. In addition, owing to the constraints of traditional concepts, lack of awareness, and policy barriers, the advantages of physical therapy have not been fully utilized in postpartum care. Therefore, enhancing participation in physical therapy in the third trimester, improving the practice of postpartum care, and promoting policy changes are issues that need to be deeply considered in the future.

## Author contributions

**Writing – original draft:** Jiangchun Zhang, Tingting Pang, Junjie Yao, Ailin Li, Li Dong, Yueting Wang, Yufeng Wang.

**Writing – review & editing:** Jiangchun Zhang, Tingting Pang, Yufeng Wang.
